# Social communities in gacha gaming and adolescent problem gambling risk: a mixed methods study

**DOI:** 10.3389/fpsyt.2026.1907023

**Published:** 2026-10-02

**Authors:** Ashley Du, Janet Wolfe

**Affiliations:** Conestoga High School, Berwyn, PA, United States

**Keywords:** adolescents, free-to-play, gacha games, loot boxes, mixed methods, problem gambling, social communities

## Abstract

**Introduction:**

Gacha games are free-to-play mobile games featuring randomized-reward mechanics to acquire characters and items. These games have grown into a multi-billion-dollar global industry, yet their relationship with adolescent problem gambling remains largely unstudied.

**Methods:**

This mixed methods study examined the extent to which gacha game spending predicts problem gambling severity among adolescents (RQ1) and how social gacha game communities influence spending behavior (RQ2). A survey of 265 adolescent gacha players recruited from online gacha gaming communities on Reddit used the DSM-IV-Multiple Response-Juvenile (DSM-IV-MR-J) to measure problem gambling severity alongside gacha spending, traditional gambling participation, and demographic covariates. Thematic analysis was conducted on 77 open-ended survey responses and 20 follow-up interviews.

**Results:**

Three quantitative analyses (Spearman's rank correlation, Mann–Whitney U test, and Firth's penalized logistic regression) found no significant relationship between gacha spending and problem gambling severity, contrasting with prior loot box research reporting significant associations. Given the low prevalence of at-risk and problem gambling in the sample, this finding should be interpreted as preliminary. Qualitative findings revealed that while social communities can amplify spending through fear of missing out (FOMO), normalization, and competitive pressure, community discouragement of spending and free-to-play (F2P) identity emerged as meaningful counterweights, reported by 13 out of 20 interview participants.

**Discussion:**

These findings raise the possibility of heterogeneous pathways among adolescent gacha players, in which some experience gacha spending as fandom-oriented and socially regulated, while others remain vulnerable to community dynamics that amplify risk. Given gacha's rapidly growing global market and the varied international regulatory responses it has received, understanding how spending behavior and community dynamics shape adolescent gambling-related risk holds increasing real-world relevance.

## 1 Introduction

Video game popularity has grown steadily over the last few decades, and online gaming has transformed the lifestyles and behavioral patterns of many adolescents and young adults by providing an easily accessible source of digital entertainment and global social interaction (1). Alongside this growth, there has been a shift in popularity from traditional home consoles, such as PlayStation and Xbox, to smartphone-based mobile video gaming. This shift has largely been due to smartphones’ portability, convenience, and increasing storage capabilities (2). Mobile games are especially easy for adolescents to access, particularly through the “freemium” or free-to-play (F2P) model, in which the core game is free to download and play but generates revenue through optional in-game purchases for cosmetics items (like skins) or convenience features (like faster progression) (2).

One prevalent type of mobile F2P game is the gacha game. The term *gacha* comes from *gachapon*, a Japanese vending machine that dispenses randomized toy rewards. Similarly, gacha games allow players to spend in-game currency to “pull” or “wish” for randomized virtual characters or items, each with different probabilities of being obtained. Outcomes are determined by random number generation rather than player skill, although some games incorporate “pity systems” that guarantee a reward after a repeated number of unsuccessful pulls. When a player runs out of currency, they can choose to purchase more with real money to continue wishing for desirable items, which may help them progress through the game more quickly and are sometimes only available for limited durations. Gacha games first emerged in Japanese mobile games around 2011 and have since become a major force in the global mobile market (3). According to industry projections, the global gacha games market value was estimated to be 19.4 billion USD in 2023 and is projected to reach 43.2 billion USD within the next decade (4), reflecting the widespread adoption of these games.

However, the randomized-reward system of gacha introduces an element of chance that gives rise to concerns about how such mechanisms may promote gambling behaviors among players. As gaming and gambling have continuously converged with the rise of digital technology, distinguishing between the two has become increasingly difficult (5). Furthermore, adolescent gamers are an especially vulnerable group for developing problem gambling due to lower levels of impulse control (6, 7). In countries such as the United States, the United Kingdom, and Australia, adolescents are not legally permitted to participate in traditional gambling activities. Nonetheless, gacha games remain accessible for children as young as 12 to download and play. As a result, policymakers, healthcare professionals, and parents have growing concerns about gacha’s potentially negative effects on adolescent gambling risk. This study examines this risk among adolescent gacha players recruited through online gacha gaming communities, described further in the Materials and Methods section. Of particular interest is the role that these online communities may play in shaping these behaviors, both as potential risk factors and, as this study suggests, as sources of protective social norms.

## 2 Literature review

### 2.1 Gacha game design and player motivations for spending

The design of gacha games often prioritizes maximizing player engagement to increase spending motivation. Lakić et al. (3) contended that gacha games exploit players’ emotional states, including feelings of luck, frustration, and anticipation of reward, to encourage repeated spending. Their findings further identify specific design elements that promote this behavior, such as limited-time characters and items released through “banners,” as well as temporary increases in winning probability, which all create urgency and scarcity. Lakić et al. maintained that even with self-regulation in place, compulsive spending cycles continue to worsen players’ well-being.

While Lakić et al. (3) primarily focused on systematic design features, other scholars have explored which specific elements of gacha games most strongly influence player spending. Woods (8) provided a complementary perspective, arguing that spending compulsions are driven by the aesthetic appeal of characters and the emotional connections players form with them. Through in-depth interviews, Woods suggested that players use characters to fulfill needs for visual satisfaction, fantasy, and self-expression. Tang et al. (9) extended this discussion by examining the social quality of life of gacha players in relation to problem gambling risk, proposing that strong social identities within gacha communities, where ownership of rare in-game items confers status, can normalize excessive spending. Through online forums, players frequently share their character draws and receive praise or sympathy, reinforcing spending norms (9).

While Lakić et al. (3) highlighted the structural features of gacha systems that drive spending, Woods (8) and Tang et al. (9) emphasized the psychological and social aspects, respectively. However, how community dynamics actually shape spending behavior among adolescents remains underexplored.

### 2.2 Gacha games and loot boxes: structural similarities and key distinctions

Gacha games share a closely related structure with loot boxes, digital containers that players open to receive random virtual rewards (10). Both systems rely on chance-based reward mechanisms. However, Koeder et al. (11) identified key differences: items from loot boxes often enhance gameplay but are not always essential to progression, whereas gacha games typically center progression around acquiring characters, making the gacha mechanic integral to both gameplay and revenue. Additionally, gacha mechanics are most prevalent in mobile games and dominant across Asian markets, while loot boxes are more common in Western PC and console titles (7, 11).

Treating gacha games and loot boxes as interchangeable may obscure meaningful differences in players’ spending motivations and patterns. Recognizing both their similarities and differences is therefore essential when evaluating their potential to contribute to problem gambling risk. Given the structural similarities, loot box research provides the most established methodological framework for examining gacha spending and problem gambling risk, while the distinctions between these systems suggest that gacha games warrant dedicated investigation rather than an assumption that loot box findings directly apply.

### 2.3 Gambling-like mechanics in loot boxes and gacha games and adolescent problem gambling risk

The advent of loot boxes and gacha game mechanics that share striking similarities with traditional gambling forms has raised concern, especially for young players. King et al. (12) found that internationally, approximately 40%–70% of adolescents had gambled in the past year and that smartphones have increased young people’s access to simulated gambling activities, potentially forming habitual patterns that create problem gambling during adolescence or adulthood. Similarly, Emond and Griffiths (13) shared this point of view, noting that adolescents’ cognitive immaturity, as well as their susceptibility to peer and marketing influences, causes them to be especially receptive to gambling-like online games.

Loot boxes have been identified as one such gambling-like mechanism. Drummond and Sauer (6) found that loot boxes meet established structural and psychological criteria for gambling, noting that all games studied allowed underage players to interact with these systems, and that early exposure to gambling is associated with heightened risk of developing problem gambling behaviors later in life. Similarly, gacha-specific structural analysis has identified a measurable reward-frequency threshold beyond which these systems exhibit gambling-like dynamics and risk fostering addiction-like compulsion, although this analysis was not adolescent-specific and does not establish direct evidence of causal changes in player behavior (14).

Gacha rewards, however, cannot be exchanged for real money and hold no official monetary payout value, unlike other forms of gambling. Nevertheless, the randomized-reward mechanics of gacha and loot boxes have drawn international attention. In 2012, Japan’s government issued a legal opinion classifying virtual items as “prizes” under the Act against Unjustifiable Premiums and Misleading Representations, leading to a ban on “kompu gacha,” a mechanic requiring players to collect items in a set for a rarer reward. Japan’s game industry has since adopted self-regulatory guidelines recommending probability disclosure and a spending threshold beyond which players may freely select a reward (15). China’s 2016 legislation required publishers to disclose the appearance rates of virtual items and banned the direct sale of randomized “treasure box” features, although some publishers have used workarounds such as labeling loot boxes as a “gift” accompanying a currency purchase (15). Most strictly, a 2018 Belgian Gaming Commission report concluded that paid loot boxes meet the legal definition of a game of chance under the Gaming and Betting Act, subjecting them to existing gambling prohibitions and criminal penalties, including enhanced penalties for violations involving minors (15).

The World Health Organization has noted that gambling-like elements in online games, such as loot boxes and microtransactions, may increase the risk of migration toward traditional gambling (16). The long-term effects of engaging with gambling-like mechanics during adolescence remain mixed, however. While Rahman et al. (17) and Potenza et al. (18) both found that earlier gambling onset is associated with higher problem gambling severity, Delfabbro et al. (19) did not find gambling at 15–16 years to be a risk factor for future problems. Despite these mixed longitudinal findings, adolescent gambling represents a concern in its own right. Evidence suggests that some adolescents already use the term “gambling” broadly enough to encompass gacha and loot box spending alongside traditional forms of gambling (20). Given adolescents’ increased vulnerability to gambling-like mechanics, gacha games warrant closer examination with regard to adolescent problem gambling.

### 2.4 Associations between loot box spending and adolescent problem gambling

Extensive research has found that loot box spending is associated with higher problem gambling severity. Brooks et al. (21) first investigated this relationship for adults by developing the Risky Loot Box Index (RLI) and demonstrating significant associations with problem gambling. Building on this work, Hing et al. (10)Zendle et al. (22), and Primi et al. (23) all found that adolescent players who spent more on loot boxes had higher problem gambling severity ratings. Rockloff et al. (24) observed similar associations among both adolescents and adults, broadening the generalizability of these findings. Palmer et al. (25) further strengthened this evidence through a longitudinal study, finding that loot box spending and RLI scores predicted later gambling initiation and expenditure. Taken together, these findings point to a positive relationship between loot box spending and problem gambling levels.

Despite this extensive research on loot boxes, comparatively little attention has been given to gacha games and adolescent problem gambling. Existing studies have often examined gacha games and loot boxes in tandem (1) or focused primarily on adult populations. Even research linking gacha game spending and adult problem gambling has had methodological limitations. For example, although Tang et al. (7) reported a positive association, Xiao et al. (26) later noted that their use of a modified measurement of problem gambling compromised the validity of the study’s findings. In addition to Tang et al. (7), the only other study identified that examined gacha spending specifically was a preprint by Han (27). Thus, a gap in knowledge remains regarding how gacha game spending relates to problem gambling risk specifically for adolescents.

The design of gacha games sustains player engagement through limited-duration content, aesthetic appeal, and emotional character attachment (3, 8), while social community dynamics may further shape spending behavior (9). Previous research has established adolescents’ vulnerability to gambling-like mechanisms (6, 13) and a consistent positive association between adolescent loot box spending and problem gambling severity (10, 22, 23). However, no comparable research has established whether this relationship extends to adolescent gacha game players. Since gacha games utilize a highly similar randomized-reward system to loot boxes while still maintaining key distinctions from them (7, 11), they necessitate focused investigation. Accordingly, this study aims to answer the following:

RQ1. To what extent does gacha game spending predict adolescents’ risk of problem gambling?

RQ2. How do adolescent gacha game players perceive the role of social gacha communities in motivating in-game spending?

Based on existing loot box research, it is hypothesized that greater gacha game spending will be associated with increased problem gambling risk. The qualitative findings of RQ2 are intended to deepen understanding of this relationship. This research aligns with United Nations Sustainable Development Goal 3 (Good Health and Well-being) by examining an emerging area of adolescent digital consumption with implications for mental health.

## 3 Materials and methods

### 3.1 Study design

This study utilized a two-part mixed methods design to address both research questions. A mixed methods approach was chosen to identify whether there is a measurable relationship between adolescents’ gacha spending and problem gambling severity (RQ1) through quantitative analysis, while also capturing players’ perspectives on social community influences on spending (RQ2) through qualitative methods.

As shown by previous studies examining in-game spending (3, 10, 22–24), a widely distributed survey is the established method for collecting the data required for statistical analysis. The first part of this study therefore consisted of a survey collecting primarily quantitative data to address RQ1, along with open-ended qualitative responses to address RQ2. The second phase consisted of optional follow-up interviews conducted via email with participants who opted in after completing the survey, using open-ended questions to explore the role of social gaming communities in motivating gacha spending. This sequential design allowed qualitative findings to build upon and contextualize quantitative results from survey data.

### 3.2 Participants

Participation was restricted to gacha game players under the age of 18, in line with the study’s focus on adolescent spending behaviors. Participants were recruited through posts on Reddit, a social news and discussion platform organized into interest-based communities known as subreddits. Reddit was selected because previous studies examining loot box and gacha mechanics have similarly recruited participants through dedicated game subreddits (2, 3, 22), and it offered direct access to the target population at no cost, supporting the study’s goal of collecting a sufficiently large sample for quantitative analysis. However, recruiting through Reddit introduced self-selection bias and uneven game representation, as subreddit engagement varies across games, and response authenticity could not be fully verified despite an age-screening question. In total, 311 participants completed the online survey, and 20 follow-up interviews were conducted.

### 3.3 Measurements

The survey utilized measurement tools similar to those used in Hing et al. (10) and Zendle et al. (22), aiming to recreate their examination of loot box spending and problem gambling severity within the context of adolescent gacha players. The survey began with an age-screening question, only allowing respondents who selected “Below 18” to continue.

The independent variable, gacha game spending, was measured through questions assessing how recently players had spent real-world money on gacha pulls, with response options matching the recency time frames used in prior adolescent loot box research (10). Before answering, participants were given a description of gacha games along with images of common wishing systems from popular titles to ensure familiarity with the mechanism being studied. A clear distinction was made between gacha games and loot boxes to ensure that data reflected spending strictly for gacha games. Players who reported spending “In the last 7 days” or “In the last 4 weeks” were classified as recent gacha spenders and were asked to report their past-month spending amount, with responses converted to USD using exchange rates shown in **Table 1** (28). Any values that lacked specification of currency were assumed to be in USD, as the example amount given in the question was “$100 USD.” Players who reported gacha spending were also asked open-ended questions exploring why they chose to spend and how social gaming communities motivate their spending behavior, providing qualitative data to address RQ2.

**Table 1:** Exchange rates used to convert reported gacha spending to USD.

Currency	Currency code	Exchange rate to USD
US dollar	USD	1.00
Australian dollar	AUD	0.67
Great British pound	GBP	1.35
Euro	EUR	1.18
Canadian dollar	CAD	0.73
Hong Kong dollar	HKD	0.13
New Zealand dollar	NZD	0.58
Chilean peso	CLP	0.0011

Exchange rates were obtained from the US Department of the Treasury’s Fiscal Data currency exchange rate tables. Rates reflect values published for December 31, 2025.

The dependent variable, problem gambling severity, was measured using the DSM-IV-Multiple Response-Juvenile (DSM-IV-MR-J), a nine-item screening tool developed by Fisher (29) to measure adolescent problem gambling and commonly used in youth gambling research in Australia and the United Kingdom (10, 12). Participants scoring 2–3 were classified as “at-risk” gamblers, and those scoring 4 or above were designated “problem” gamblers.

Participation in the 11 monetary gambling activities listed in **Table 2** was measured following Hing et al. (10), allowing the relationship between gacha spending and problem gambling severity to be examined while controlling for involvement in traditional gambling. Participants who indicated participating in any activity “In the last 7 days” or “In the last 4 weeks” were considered recent participants in monetary gambling. Finally, demographic information including age, gender, and race/ethnicity was collected for use as covariates. Gender was coded as 1 = male, 2 = female, 3 = gender diverse, 4 = prefer not to say, with male as the reference category for regression analyses. The full survey instrument is provided in **Supplementary Data Sheet 1** to allow for transparency and replication.

**Table 2:** Monetary gambling activities assessed in the survey.

Gambling activity
Lottery games
Pokies/slot machines (electronic gaming machines)
Race betting
Keno (lottery-style number game)
Sport betting
Casino games
Bingo (played for money)
Poker
Esports betting
Fantasy sports betting
Private betting

For each activity, participants indicated their participation using the following response options: “In the last 7 days,” “In the last 4 weeks,” “In the last 12 months,” “More than 12 months ago,” and “Never.” These time frames were consistent with prior adolescent gambling research (10) and allowed for classification of recent participation.

The follow-up interviews consisted of 10 open-ended questions distributed via email, focusing on the role of social gaming communities in influencing spending decisions and including prompts such as “Do you feel that being part of these communities influences how you think about spending money in the game? Why or why not?” All interview questions are provided in **Supplementary Data Sheet 2**.

### 3.4 Procedure

The survey was created using Google Forms and posted to the 14 subreddits for popular gacha games listed in **Supplementary Data Sheet 3**. The survey remained open from January 18, 2026, to February 9, 2026. Each post stated that only adolescents were eligible to participate, and posts were staggered and published on Saturday evenings to avoid being flagged as spam and to maximize visibility. Of the 311 respondents, 56 opted in for a follow-up interview, and questions were emailed 4 weeks after the initial survey post, gathering 20 responses.

### 3.5 Ethical considerations

All survey and interview questions were reviewed and approved by the Conestoga High School Institutional Review Board, constituted in accordance with the College Board’s AP Research requirement that all student research involving human participants be reviewed prior to data collection. The panel evaluates proposed studies for participant risk, informed consent procedures, and data privacy protections. Participation was entirely voluntary. Prior to starting the survey, all participants were provided with a clear description of the study’s purpose, the researcher’s identity and contact information, and an explanation of how their data would be used. Participants provided explicit informed consent before moving forward and were notified they could withdraw at any time without penalty. All survey and interview responses were anonymous, with no identifying information collected that could link responses to participants. Contact information provided for interviews was collected solely to distribute and receive responses and stored separately from survey data, ensuring that interview participation could not be linked to individual survey answers.

Parental consent was not obtained, as requiring parental verification would have necessitated collecting identifying information from participants recruited through a broadly distributed, anonymous online survey, compromising the complete anonymity the study’s design depended upon. The study was strictly observational and non-interventional, meaning no experimental manipulation was used, posing minimal risk to participants. This approach was confirmed as acceptable by the AP Research instructor and is consistent with comparable adolescent loot box research in which participants were recruited via Reddit and informed consent was gathered directly from adolescent participants without documented parental consent procedures (22). Participant safeguarding was managed through complete anonymity, informed consent, and voluntary participation; however, the absence of proactive mental health and problem gambling support resources at the conclusion of the survey is acknowledged as a limitation of the safeguarding protocol.

### 3.6 Data analysis

Following the non-parametric approach of Zendle et al. (22) and the covariate-controlled approach of Hing et al. (10), the relationship between gacha game spending and problem gambling severity was analyzed through three complementary analyses. First, a Spearman’s rank correlation determined whether spending amount was associated with DSM-IV-MR-J scores. Second, a Mann–Whitney U test compared DSM-IV-MR-J scores between recent and non-recent gacha spenders. Both non-parametric tests were selected because gacha spending amount and DSM-IV-MR-J total scores exhibited significant positive skew, and the two analyses were conducted using JASP (Version 0.96).

Third, given the severe positive skew in DSM-IV-MR-J scores and the very low prevalence of at-risk or problem gambling in the sample (n = 7, 2.6%), standard multiple linear regression was deemed inappropriate due to insufficient outcome variance for reliable estimation. Instead, Firth’s penalized logistic regression (30) was conducted to examine whether gacha spending amount predicted elevated gambling risk (DSM-IV-MR-J score ≥2, combining at-risk and problem gambling classifications) while controlling for age, gender, and traditional gambling participation, using the logistf package (Version 1.26.0) in R (Version 4.6.1). Firth’s penalization was selected because it produces reliable, bias-reduced estimates for binary outcomes with very low prevalence and handles complete separation, which occurred because all seven participants meeting the elevated risk threshold were male (31, 32). The model converged successfully, as indicated by the absence of convergence warnings from the logistf package.

Thematic analysis followed an inductive approach, allowing themes to emerge from the data without predetermined categories, following the approach of Braun and Clarke (33). Coding was conducted by the lead researcher; as a single coder was used, no inter-coder discrepancies arose. As an active gacha game player, the lead researcher brought contextual familiarity with gacha community culture, terminology, and mechanics, which facilitated recognition of culturally significant patterns in participant responses, including the social significance of F2P identity, or the status of a gacha player who progresses through the game without spending any real money, as a community norm rather than merely an absence of spending. Reflexivity was maintained throughout the analytical process, with the lead researcher actively interrogating how personal experience as a gacha player may have shaped theme identification and interpretation.

## 4 Results

### 4.1 Data cleaning

A total of 311 responses were collected. Forty-three were excluded for failing the age-screening question, and three additional responses were excluded due to evidence of insincere responding, producing a final analysis sample of 265 respondents (85.2% of the original sample).

### 4.2 Descriptive results

Participants ranged in age from 13 to 17 years (M = 15.79, SD = 1.28). Gender was distributed across male (40.8%), female (35.5%), gender diverse (14.7%), and prefer not to say (9.1%) categories. Full demographic characteristics are presented in **Table 3**. Gacha spending patterns are summarized in **Table 4**. Forty-three participants (16.2%) were classified as recent gacha spenders, with past-month expenditure varying considerably (M = $58.81 USD, Mdn = $30.00, range = $5.00–$402.00).

**Table 3:** Demographic characteristics of the sample (N = 265).

Characteristic	n	%
Age		
M (SD)	15.79 (1.28)	—
Range	13–17	—
Gender		
Male	108	40.8
Female	94	35.5
Gender diverse	39	14.7
Prefer not to say	24	9.1
Hispanic, Latino, or Spanish origin		
Yes	29	10.9
No	236	89.1
Race		
Asian or Asian American	129	48.7
White	91	34.3
Multiracial	22	8.3
Black or African American	13	4.9
Other or Unknown	10	3.8

Gender diverse includes non-binary and all other non-male/non-female gender identities. Hispanic/Latino ethnicity was assessed separately from race, consistent with US Census Bureau standards.

**Table 4:** Gacha game spending characteristics (N = 265).

Variable	n	%
Gacha spending recency		
Recent spender (last 7 days or last 4 weeks)	43	16.2
Non-recent spender (never or more than 4 weeks ago)	222	83.8
Spending amount (USD) among recent spenders		
n	43	
Mean	$58.81	
Median	$30.00	
Range	$5.00–$402.00	

Spending amounts originally reported in non-USD currencies were converted to USD using exchange rates in **Table 1**. Recent spenders are defined as participants who reported spending real-world money on gacha pulls within the last 7 days or last 4 weeks.

DSM-IV-MR-J total scores are summarized in **Table 5**. Scores were notably low across the sample (M = 0.12, SD = 0.73), with 97.4% of the participants classified as non-problem gamblers. Only four participants (1.5%) met the criteria for at-risk gambling and three (1.1%) for problem gambling. Given that so few participants met the criteria for at-risk or problem gambling, continuous DSM-IV-MR-J total scores were used for Spearman’s correlation and Mann–Whitney U test, while a binary threshold of a DSM-IV-MR-J score ≥2, combining at-risk and problem gambling classifications, was used for Firth’s penalized logistic regression to address the low event prevalence and resulting estimation bias.

**Table 5:** DSM-IV-MR-J scores and problem gambling classification.

Variable	n	%
Problem gambling classification		
Non-problem (score 0–1)	258	97.4
At-risk (score 2–3)	4	1.5
Problem gambler (score 4+)	3	1.1
DSM-IV-MR-J total score		
Mean (SD)	0.12 (0.73)	
Range	0–7	

The DSM-IV-MR-J is a nine-item screening tool scored 0–9. Scores of 2–3 indicate at-risk gambling, and scores of 4 or above indicate problem gambling (29).

Traditional monetary gambling participation was similarly rare and can be seen in **Table 6**, with only 11 participants (4.2%) reporting recent involvement in any of the 11 gambling activities that were assessed (M_activities_ = 0.09, range = 0–8).

**Table 6:** Traditional monetary gambling participation.

Variable	n	%
Recent gambling participation		
Any recent gambling activity (last 7 days or last 4 weeks)	11	4.2
No recent gambling activity	254	95.8
Number of gambling activities (0–11)		
Mean	0.09	
Range	0–8	

Recent gambling participation was defined as involvement in any of the 11 assessed monetary gambling activities within the last 7 days or last 4 weeks, consistent with Hing et al. (10).

### 4.3 RQ1: gacha spending and problem gambling severity

Three analyses were conducted to examine the relationship between gacha game spending and problem gambling severity. Key results are summarized in **Table 7**.

**Table 7:** Summary of statistical analyses examining the relationship between gacha spending and problem gambling severity.

Analysis	Statistic	Effect size	p
Spearman’s correlation	r_s_ = .060	η^2^ = .004	.330
Mann–Whitney U	U = 4611	r = .034, 95% CI [−0.154, 0.219]	.310
Firth’s penalized logistic regression (overall model)	χ²(6) = 26.00	—	<.001

Dependent variable for Spearman’s correlation and Mann–Whitney U analyses is continuous DSM-IV-MR-J total score. Dependent variable for Firth’s penalized logistic regression is binary at-risk or problem gambling status (DSM-IV-MR-J score ≥2). η² for Spearman’s correlation was calculated by squaring Spearman’s r value.

#### 4.3.1 Spearman’s correlation

A Spearman’s rank correlation was conducted to examine whether gacha spending amount was associated with problem gambling severity. As seen in **Table 7**, there was no significant correlation between the two variables (r_s_ = .060, p = .330, η² = .004). The observed effect size was well below the minimum threshold of η² = .03 predetermined as meaningful by Zendle et al. (22) in comparable loot box research, suggesting that the relationship between gacha spending and problem gambling severity in this sample was negligible.

#### 4.3.2 Mann–Whitney U test

A Mann–Whitney U test was conducted to examine whether recent gacha spenders differed from non-recent spenders in problem gambling severity. As seen in **Table 7**, no significant difference was found between recent spenders (n = 43) and non-recent spenders (n = 222), with both groups having a median score of 0 (U = 4611, p = .310, 95% CI [−0.154, 0.219]). The confidence interval included zero, indicating that the true difference could possibly be zero or even negative.

#### 4.3.3 Firth’s penalized logistic regression

To further examine the relationship between gacha spending and problem gambling severity while controlling for potential confounding variables, Firth’s penalized logistic regression was conducted to predict at-risk or problem gambling status (DSM-IV-MR-J score ≥2) from gacha spending amount, age, gender, and traditional gambling participation. At-risk and problem gambling classifications were combined into a single binary outcome due to insufficient cell sizes for separate analysis (at-risk, n = 4; problem, n = 3). The overall model was statistically significant [likelihood ratio χ²(6) = 26.00, p <.001], with detailed results presented in **Table 8**. However, gacha spending amount, age, and gender were not significant predictors of at-risk or problem gambling status. Traditional gambling participation was the only significant predictor (OR = 3.367, 95% CI [1.527, 11.737], p <.001), indicating that each additional traditional gambling activity was associated with 3.4 times greater odds of meeting the at-risk or problem gambling threshold, which is consistent with prior research and suggests that the DSM-IV-MR-J functioned as expected for this sample.

**Table 8:** Firth’s penalized logistic regression predicting at-risk or problem gambling status (DSM-IV-MR-J score ≥2) (N = 265).

Predictor	OR	95% CI	p
Gacha spending amount (USD)	1.007	[0.983, 1.021]	.290
Age	1.410	[0.672, 4.973]	.407
Traditional gambling count	3.367	[1.527, 11.737]	<.001
Gender (female vs. male)^a^	0.154	[0.001, 1.523]	.122
Gender (gender diverse vs. male)^a^	0.336	[0.002, 3.423]	.412
Gender (prefer not to say vs. male)^a^	0.424	[0.001, 4.276]	.530

Wide confidence intervals for gender variables reflect complete separation: no female, gender diverse, or prefer not to say participants met the elevated risk threshold.

OR, odds ratio.

^a^Male was used as the reference category for gender comparisons.

### 4.4 RQ2: Thematic analysis

Thematic analysis of 42 survey responses on spending motivations (Q1), 35 survey responses on social community influences (Q2), and 20 follow-up interviews identified five themes regarding how social communities shape adolescent gacha players’ in-game spending behavior: 1) character attachment and aesthetic appeal as primary spending motivations, 2) fear of missing out (FOMO) amplified by community discussions, 3) community normalization of spending, 4) social comparison and competitive pressure, and 5) community discouragement of spending and F2P identity.

#### 4.4.1 Theme 1: character attachment and aesthetic appeal

The most dominant theme across all data sources was that players primarily spend to obtain specific characters they feel emotionally attached to or find visually appealing, rather than due to direct social influence. This motivation appeared in 27 Q1 responses, 4 Q2 responses, and 12 interview responses. As one participant described, “Collecting my favorite characters both in-game and in real life (merch) is how I feel I can express my love for them” (Survey Respondent 45). Another noted, “It’s the only way I can get cards/characters I like … I’m not a big fan of the gambling process at all, I just want the pretty cards/characters” (Survey Respondent 165). Interview participants echoed this statement, with one stating, “I don’t mind spending in Genshin because the game means a lot to me. I’ve been playing for about 4–5 years and the <$100 I’ve spent feels like the least I can do to support a game that’s given me community and thousands of hours of entertainment” (Interview 9).

#### 4.4.2 Theme 2: FOMO amplified by community discussions

Five Q1 responses, three Q2 responses, and six interview participants reported that community discussions surrounding limited-time banners and hyped characters intensified FOMO, in turn motivating spending. One survey respondent noted, “I don’t like missing out on limited-time character cards, and seeing other people having them make me wanna have them as well, it’s basically the FOMO” (Survey Respondent 138). Interview participants described similar experiences, with one explaining that community discussions about must-have characters created pressure: “when a new limited character or weapon gets implemented there will be a discussion about how important or must-have this new character or weapon is, which can lead to fomo (fear of missing out)” (Interview 15). Another participant described how seeing community hype around a character directly led to spending: “I saw people on the subreddit posting videos of her gameplay and fanart of her character, and I really really wanted her. So I dropped $30 to get her” (Interview 7).

#### 4.4.3 Theme 3: community normalization of spending

Five interview participants noted that observing widespread spending within communities made spending feel more acceptable and normal. One interview participant stated plainly that in their community, “spending money is normalized, even if it is not required or explicitly suggested” (Interview 20). Another noted that seeing many people spend “makes paying real money in games seem normal” (Interview 14). A third participant observed that communities with many whales, or players who spend large amounts of money on gacha (3), make spending more expected: “if the community is filled more with spenders/whales, then I might be inclined to spend” (Interview 11).

#### 4.4.4 Theme 4: social comparison and competitive pressure

Five Q1 and seven Q2 responses described how community discussions about gameplay performance and account quality created social comparison dynamics that motivated spending. One survey respondent described how satisfaction diminished upon seeing others with a stronger version of the same character: “you can initially want a single copy of a character to enjoy it and quickly fall out of satisfaction when you learn not only do most people already have it, but they have it on a higher promotion level than you” (Survey Respondent 120). Interview participants described more explicit competitive pressure, with one noting that communities will “collectively agree that if you don’t have certain character or weapon your account is bricked (not worth to continue)” (Interview 15), and another describing how performance comparisons in Honkai Star Rail motivated a $200 purchase after seeing “the difference in damage and performance between E0 and E2 was almost 300%” (Interview 17).

#### 4.4.5 Theme 5: community discouragement of spending and F2P identity

A significant counterweight to the above themes emerged across multiple sources: community discouragement of spending or absence of community influence was the most prevalent theme in Q2 responses (11/35 respondents), and 13 out of 20 interview participants described communities that discouraged spending or expressed pride in F2P identity. One interview participant described how Reddit communities “actually recommend not spending impulsively on gacha games” and that these reminders “have often persuaded me not to needlessly pull for characters” (Interview 5). Another noted that “free to play is seen as more impressive than pay to play” (Interview 20), while a third described an informal competition where players “make sure us normal players can outshine the P2W [pay to win] players without spending money, to prove a point” (Interview 6). One survey respondent explained this dynamic particularly clearly, noting that the F2P identity acts almost like “a streak, where you play the game without using money no matter how tempting it is” (Interview 1).

## 5 Discussion

### 5.1 Social community influence on gacha spending

Across all data sources, character attachment and aesthetic appeal emerged as the primary spending motivation, with social community factors playing a secondary amplifying role rather than independently driving spending behavior. FOMO was the most prominent community-driven spending motivator, with participants describing how community discussions surrounding limited-time banners intensified their desire to obtain characters. This trend extends the findings on limited-time mechanics of Lakić et al. (3) by demonstrating that community discussions further amplify their psychological impact on adolescent players. Community normalization of spending was also observed, consistent with research on adolescents’ susceptibility to peer influence (13). Competitive pressure emerged in games with pay-to-win elements, where community discussions about differences in character performance motivated spending.

The most striking community-specific finding, however, was the prevalence of F2P identity and community discouragement of spending, complicating existing literature’s understanding of how social communities may promote spending (9, 22). The data suggest that F2P identity operates as a multidimensional social construct rather than merely an absence of spending. Participants framed free-to-play status as a source of community prestige, suggesting a status-seeking dimension in which skill and progression without monetary investment confer social recognition. This viewpoint is consistent with research findings that players who use microtransactions are perceived as having lower skill and status in online gaming contexts (34), suggesting that abstaining from spending may carry social significance beyond being a purely financial decision. Participants also described F2P identity in terms of resistance against pay-to-win dynamics, actively competing against spending players to prove a point, positioning being free-to-play as a deliberate rejection of the game’s monetization structure. A self-control narrative also emerged, with this identity described as a streak to be maintained despite temptation, framing abstention from spending as an ongoing personal achievement. Although economic constraints may also contribute to free-to-play status for some players, as suggested by two participants who described practical barriers to spending, the predominant framing across data sources was one of chosen identity carrying social meaning through status, resistance, and self-control. Together, these dimensions suggest that F2P identity may function as a protective social norm that buffers against spending pressure through multiple psychological pathways, a pattern that has not been previously documented in gacha or loot box research. Game developers, parents, and policymakers should therefore consider the dual nature of gacha communities. While some dynamics promote spending, the prevalence of free-to-play norms suggests that community-level protective factors may already be present within these spaces.

In addition to their role in affecting spending behavior, these community dynamics carry implications for player well-being. Prior research has linked gacha spending to compulsive patterns that may worsen player well-being (3), identified structural design features that may foster addiction-like compulsions in players (14), and found that early exposure to gambling-like mechanics is associated with heightened long-term risk of developing problem gambling behaviors (6); this study’s finding that community norms can actively discourage such spending suggests that these communities may function as a protective buffer against these risks, rather than merely a neutral or risk-amplifying influence.

### 5.2 The relationship between gacha game spending and problem gambling severity

These qualitative findings offer important context for the study’s quantitative results. Gacha spending did not significantly predict adolescent problem gambling severity across all three analyses, a null finding that contrasts with prior loot box research finding significant associations between spending and problem gambling severity (10, 22). However, given the very low prevalence of at-risk and problem gambling in the sample (n = 7, 2.6%), these null findings should be interpreted as preliminary rather than as definitive evidence of an absence, as the study may have been underpowered to detect associations if they exist. When controlling for age, gender, and monetary gambling participation, traditional monetary gambling involvement was the only significant predictor of at-risk or problem gambling status (OR = 3.367, p <.001). This finding is consistent with prior literature and suggests that the DSM-IV-MR-J functioned as expected in this sample.

Adolescent players in this study were primarily motivated by character attachment and aesthetic appeal rather than the gambling process itself, and social communities were found to actively discourage excessive spending. These qualitative findings may offer one possible explanation for the preliminary null results. Overall, the findings suggest an early conceptual model of gacha-specific pathways that may differ from loot box spending. Whereas the thrill and excitement of opening randomized rewards have been identified as a distinct motivational factor in loot box spending (22), this pattern was notably absent in the current sample, where character attachment and aesthetic appeal dominated. Potential risk-amplifying features identified included FOMO driven by limited-time banners, community normalization of spending, and pay-to-win competitive pressure, while protective features included F2P identity norms and character attachment as the primary spending motivation. These findings raise the possibility of heterogeneous pathways among adolescent gacha players, in which some may experience gacha spending as fandom-oriented and socially regulated, while others remain vulnerable to FOMO, competitive pressure, normalization of spending, and pay-to-win dynamics. However, further research with larger samples and greater proportions of at-risk and problem gamblers is needed before definitive conclusions about the relationship between gacha spending and problem gambling risk can be drawn. With gacha’s rapidly growing global market and the varied international regulatory responses it has received (15), understanding how spending behavior and community dynamics shape adolescent gambling-related risk holds increasing real-world relevance.

### 5.3 Limitations

The primary limitation of this study was the small number of at-risk and problem gamblers in the sample (n = 7), limiting statistical power. Gacha spending data were self-reported, which may have introduced inaccuracies. The spending measure captured only recent past-month expenditure among recent spenders, potentially missing more clinically relevant dimensions of spending behavior. Reddit recruitment also introduced self-selection bias and uneven game representation, with the Project Sekai subreddit generating disproportionately higher responses. Importantly, recruitment through gacha game subreddits may have also overrepresented adolescents actively involved in online communities with strong F2P norms, while underrepresenting more isolated players or those with more problematic spending patterns, potentially inflating the prevalence of community protective factors observed in the qualitative findings. The thematic analysis that produced these findings was conducted by a single coder, which may have introduced interpretive bias despite reflexive precautions. The study’s cross-sectional design also means findings reflect associations and do not establish causality between gacha spending and problem gambling. Finally, proactive mental health and problem gambling support resources were not included at the conclusion of the survey, representing a limitation of the study’s safeguarding.

### 5.4 Future research

The low DSM-IV-MR-J scores observed highlight the need for gacha-specific problem spending measures to complement traditional gambling screeners, similar to the Risky Loot Box Index developed by Brooks et al. (21) for loot box spending. Existing instruments may not fully capture the unique motivations and patterns of adolescents’ gacha game expenditure, and neither the DSM nor WHO’s ICD-11 currently includes a diagnostic classification specific to gacha games. Future research should aim to recruit larger samples with greater proportions of at-risk and problem gamblers to improve statistical power, minimize self-selection bias, ensure more balanced representation across gacha game communities, and employ longitudinal designs to establish whether gacha spending predicts the development of problem gambling behaviors over time. Additionally, future work should incorporate more comprehensive spending measures capturing dimensions such as cumulative expenditure, episodic patterns, spending relative to disposable income, and access to parental payment methods, which may be more clinically relevant than absolute past-month spending amount.

## 6 Conclusion

This study found that social community influences on gacha spending are more complex than previously described: F2P identity operates as a meaningful protective factor against spending pressure in many adolescent gacha communities, a finding that has not been documented in prior literature. Gacha spending did not significantly predict problem gambling severity, although given the study’s limited statistical power, these findings should be interpreted as preliminary rather than as definitive evidence of absence. These results raise the possibility of heterogeneous pathways in adolescent gacha spending, with some players experiencing it as fandom-oriented and socially regulated, while others may remain vulnerable to risk-amplifying community dynamics. As gacha games continue to grow in global popularity among adolescents, targeted research with larger samples and improved measurement tools are needed to better understand their relationship with problem gambling risk.

## Data Availability

The datasets for this article are not publicly available due to concerns regarding participant anonymity. Requests to access the datasets should be directed to the corresponding author.
