# Supplementary material for: Social communities in gacha gaming and adolescent problem gambling risk: a mixed methods study
Source: Front Psychiatry. 2026 Oct 2;17:1907023. doi: 10.3389/fpsyt.2026.1907023 (PMC13674343; doi:10.3389/fpsyt.2026.1907023)
Supplement: Supplementary file 1 [file DataSheet1.pdf]

Appendix A

Adolescent Gacha Game Player Spending Analysis

This survey is part of a research study examining gacha game spending, social gaming communities, and gambling-related behaviors among adolescents.

* Indicates required question

The purpose of this study is to examine the correlation between gacha game spending and levels of problem gambling among adolescent gacha game players, as well as to explore how social gacha game communities may influence this relationship. This research is being conducted by [redacted]: an AP Research student at [redacted] and a gacha game player herself. If you have any questions, please contact [redacted] at [redacted]

By taking this survey, all responses will remain anonymous so neither [redacted] nor any teachers will know what your answers are. You may stop answering survey questions at any time. At the end of this survey, you may choose to volunteer for a separate, optional, and anonymous interview about gacha game spending and social gaming communities. If you choose to participate, your contact information will be collected only for scheduling purposes and will not be linked to your survey responses. Your survey answers will remain anonymous even if you choose to participate in an interview.

This research aims to inform future policymaking decisions regarding the regulation of gacha games, and may also help participants reflect on their own video game spending decisions. Thank you for spending your time to take this approximately 10 minute survey.

Before taking this survey, by clicking this box you are giving consent for your responses to be anonymously used in a research project.

Check all that apply.

- [ ] I give consent for my answers to be used in a research project

Age

1. How old are you? *

Mark only one oval.

- ( ) Below 18 — *Skip to question 3*
- ( ) 18 or above — *Skip to section 11 (Thank you for your time!)*

Gacha Game Involvement

Gacha games are video games that are free to download and play but include optional in-game purchases for virtual currency that can be used to "pull/roll/wish" for certain characters or items. When wishing, the rewards are all randomized and characters/items have different probabilities of being obtained, with no immediate guarantee that players

will get what they wish for. Popular gacha games include *Genshin Impact, Honkai: Star Rail, Arknights, Fate/Grand Order*, and more. In some cases, players may wish for free, but can also choose to spend real money to continue wishing.

For the purpose of this study, gacha is *not* the same as "loot boxes," another randomized-rewards system found in games such as *Fortnite, Overwatch 2, Apex Legends,* etc. Loot boxes are often presented as a digital chest or box that can be opened, but the content inside is usually only add-ons and is not integral to the video game itself. On the other hand, the system of wishing through gacha is central to the gameplay within gacha games. Pictures of common gacha wishing systems are shown below.

Character banner

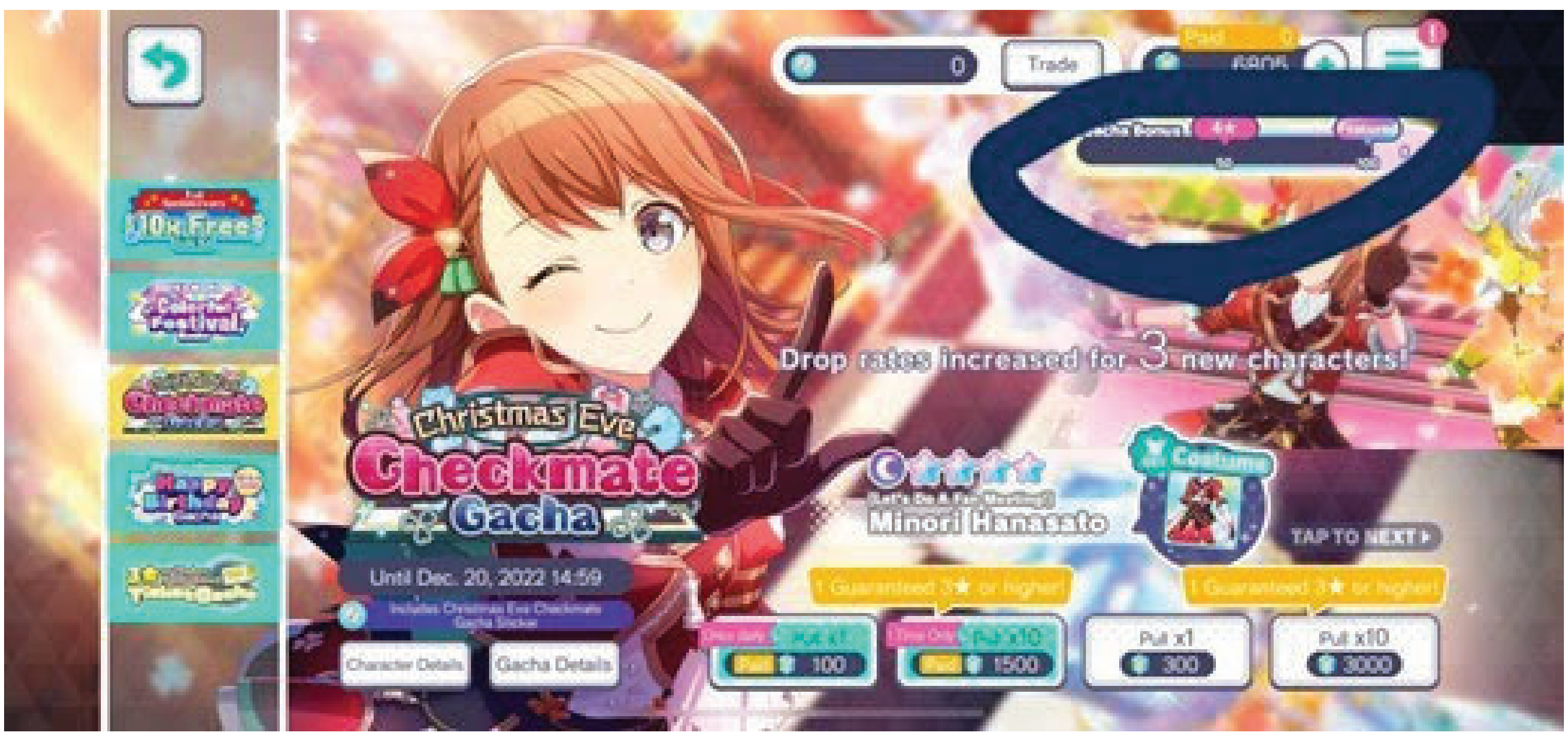

Character banner

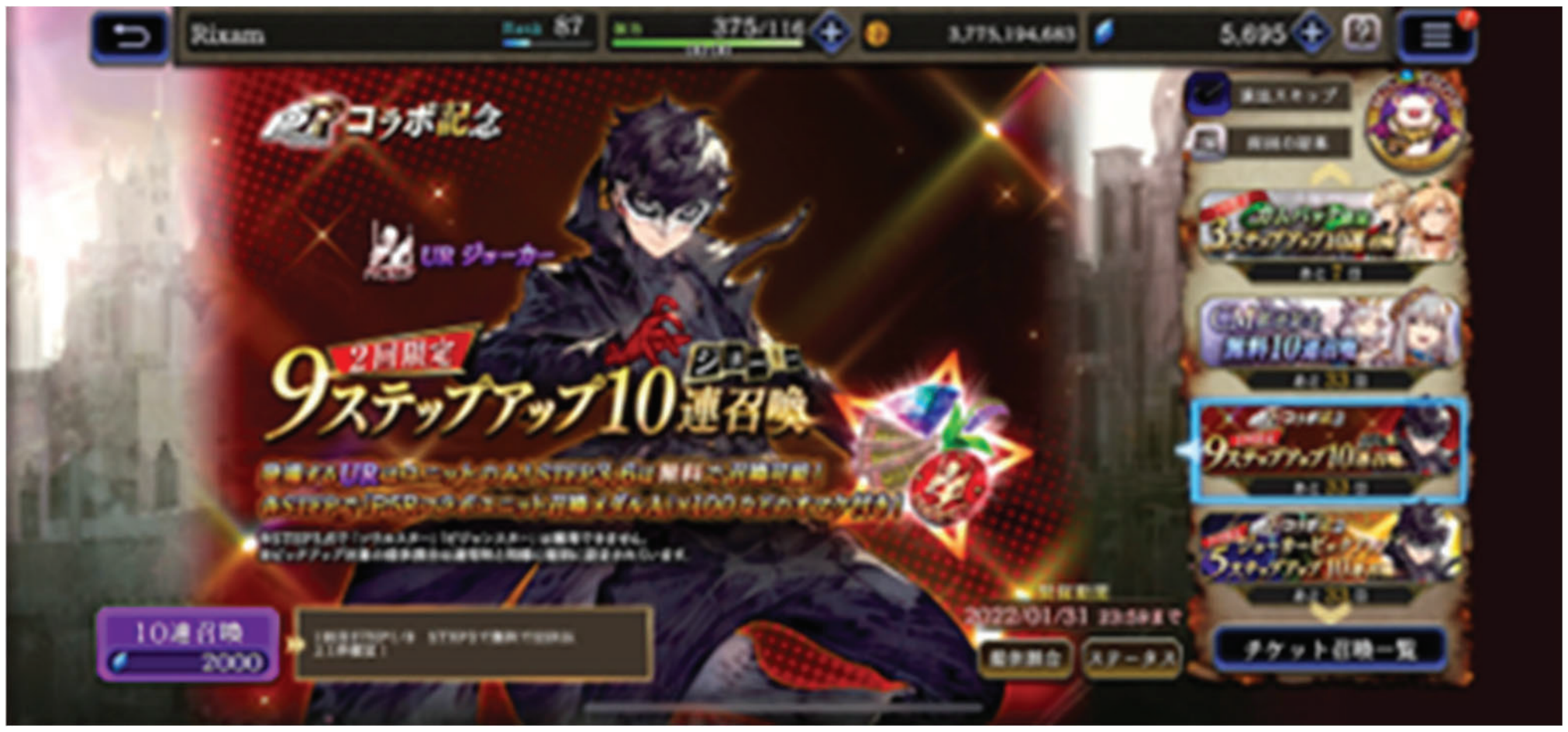

Wish results

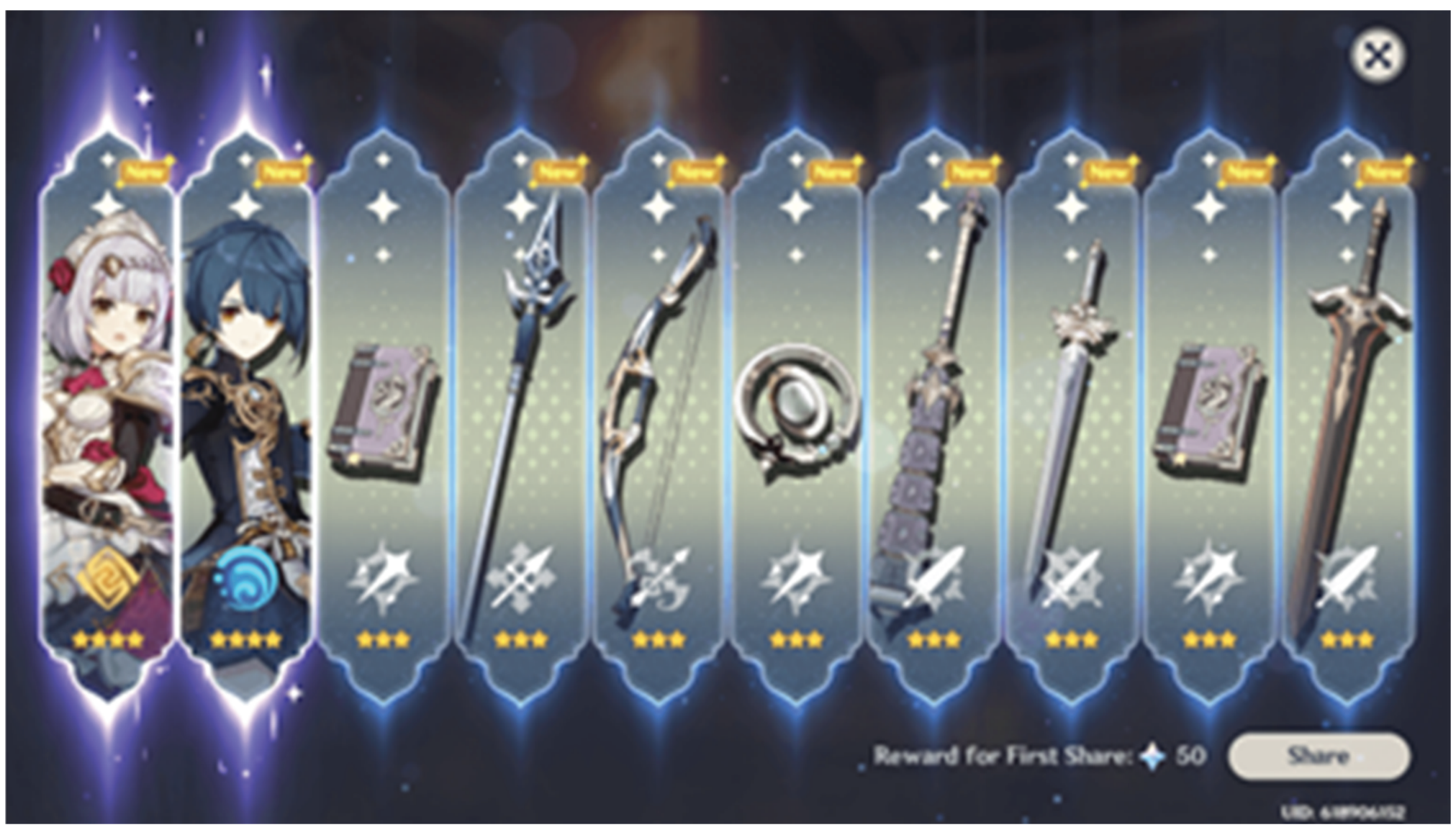

Wish results

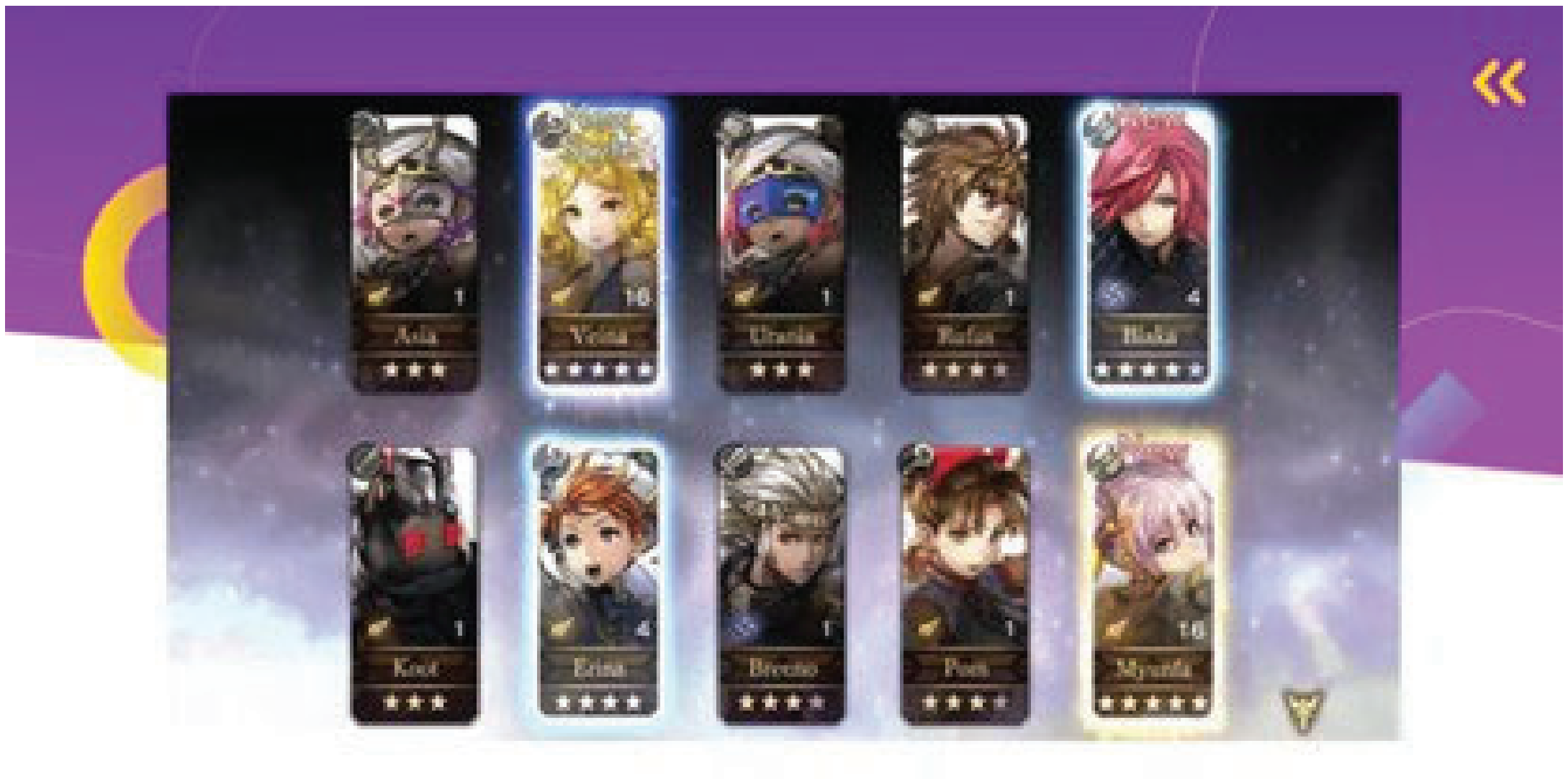

2. When, if ever, have you paid real-world money to pull/roll/wish on gacha? *

This includes paying real world money for an in-game currency that is used to buy gacha pulls/rolls/wishes.

This does *not* include spending money on "loot boxes" *or* wishing for characters without spending any real-world money (being an F2P player).

Mark only one oval.

- ⬭ In the last 7 days *Skip to question 4*
- ⬭ In the last 4 weeks *Skip to question 4*
- ⬭ In the last 12 months *Skip to question 12*
- ⬭ More than 12 months ago *Skip to question 12*
- ⬭ Never *Skip to question 12*

Gacha Game Spending

Next, we would like to gather some information about your spending habits within gacha games.

3. Which single gacha game have you spent the most money on during the past month? *

____

4. Thinking about this game, approximately how much money have you paid for gacha pulls/rolls/wishes during the past month? *
Please include what type of currency you use. Example: "$100 USD"
This includes paying real world money for an in-game currency that is used to buy gacha pulls/rolls/wishes.

____

5. Approximately how long had you played this gacha game for before the first time you paid real-world money to pull/roll/wish? *

Mark only one oval.

- ◯ Less than a day
- ◯ 1 day—1 week
- ◯ 1 week—1 month
- ◯ More than 1 month

6. When it comes to this game, can characters and/or items obtained from gacha give gameplay advantages? *

Mark only one oval.

- Yes
- No

7. When it comes to this game, does the desire to fit in or look better with certain characters and/or items motivate you to spend on gacha? *

Mark only one oval.

- Yes
- No

8. Have interactions with other players in your gacha game community ever made you feel pressure to spend money on gacha pulls/rolls/wishes? *

Mark only one oval.

- Yes
- No

9. Why would you say that you pull/roll/wish on gacha games?

10. What aspects of the social gaming community surrounding this gacha game, if any, motivate you to spend on pulls/rolls/wishes?

Gambling Behavior

We would like to gather more information about your experience with gambling-related activities. All responses are anonymous and cannot be linked back to you.

11. When, if ever, did you last participate in any form of monetary gambling <u>besides</u> gacha game spending? This includes but may not be limited to: *

1. Lottery games
2. Pokies/slot machines (electronic gaming machines)
3. Race betting
4. Keno (lottery-style number game)
5. Sport betting
6. Casino games
7. Bingo (played for money)
8. Poker
9. Esports betting
10. Fantasy sports betting
11. Private betting

This does *not* include spending money on "loot boxes" or another similar randomized-rewards system within a video game.

Mark only one oval.

- In the last 7 days *Skip to question 13*
- In the last 4 weeks *Skip to question 13*

- ⬭ In the last 12 months — *Skip to question 15*
- ⬭ More than 12 months ago — *Skip to question 24*
- ⬭ Never — *Skip to question 24*

Gambling Activities

Next, we would like to know more about which gambling-related activities you participate in. All responses are anonymous and cannot be linked back to you.

12. Which monetary gambling activities have you participated in? Check all that apply. *

    This does *not* include spending money on gacha games, "loot boxes," or another similar randomized-rewards system within a video game.

    Check all that apply.

    - [ ] Lottery games
    - [ ] Pokies/slot machines (electronic gaming machines)
    - [ ] Race betting
    - [ ] Keno (lottery-style number game)
    - [ ] Sport betting
    - [ ] Casino games
    - [ ] Bingo (played for money)
    - [ ] Poker

- [ ] Esports betting
- [ ] Fantasy sports betting
- [ ] Private betting (bets with family/friends outside of formal gambling settings)
- [ ] Other: ____________________

13. When did you last participate in this activity/these activities? *

Mark only one oval.

- ( ) In the last 7 days
- ( ) In the last 4 weeks
- ( ) In the last 12 months
- ( ) More than 12 months ago

Gambling Screen

Now we would like to ask you some questions about gambling in general. This does *not* include spending money on gacha games, "loot boxes," or another randomized-rewards system within a video game. All responses are anonymous and cannot be linked back to you.

Answer for the past 12 months only, even if you have gambled longer ago.

In the past 12 months...

14. How often have you found yourself thinking about gambling or planning to gamble? *

Mark only one oval.

- Never
- Once or twice
- Sometimes
- Often

15. How often have you gambled to help you escape from problems or when you are feeling bad? *

Mark only one oval.

- Never
- Once or twice
- Sometimes
- Often

16. Have you felt bad or fed up when trying to cut down on gambling? *

Mark only one oval.

- Never
- Once or twice
- Sometimes
- Often

17. Have you needed to gamble with more and more money to get the amount of excitement you want? *

Mark only one oval.

- Never
- Once or twice
- Sometimes
- Often

18. Have you ever spent much more than you planned to on gambling? *

Mark only one oval.

- Never
- Once or twice
- Sometimes
- Often

19. Have you ever taken money from any of the following without permission to spend on gambling: dinner money or fare money, money from family, money from things you've sold, money from outside the family, somewhere else *

Mark only one oval.

- Never
- Once or twice
- Sometimes
- Often

20. Has your gambling ever led to the following: 1) arguments with family or friends or others, 2) missing school *

Mark only one oval.

- Never
- Once or twice
- Sometimes
- Often

21. Has your gambling ever led to the following: Telling lies to family or friends or others *

Mark only one oval.

- Never
- Once or twice
- Sometimes
- Often

22. After losing money by gambling, have you returned another day to try to win back the money you lost? *

Mark only one oval.

- Never
- Once or twice
- Sometimes
- Often

Demographic Information

We would now like to gather some details about your characteristics.

23. What is your age? *

Mark only one oval.

- Under 12
- 12
- 13
- 14
- 15
- 16

17

24. What is your gender? *

Mark only one oval.

- Female
- Male
- Non-binary
- Prefer not to say
- Other: ______

25. Are you of Hispanic, Latino, or Spanish origin? *

Mark only one oval.

- Yes
- No

26. What is your race? Select all that apply. *

Check all that apply.

- [ ] American Indian or Alaska Native
- [ ] Asian
- [ ] Black or African American
- [ ] Native Hawaiian or Other Pacific Islander

☐ White

☐ Other: ______________________

Additional Information

27. The questions that you have answered during this survey are intended to help us understand motivations behind gacha game spending and determine if there is any relationship between spending on gacha games and problem gambling for adolescents. Do you have any additional comments about this study, or anything else that you would like us to know?

____

____

____

____

____

28. If you play gacha games — would you be willing to participate in a short, anonymous follow-up interview about the social dynamics of gacha game communities and how they affect your gacha spending behavior?

Mark only one oval.

- ◯ Yes *Skip to question 30*
- ◯ No

Contact Information

Please provide your email or phone number below so the researcher can contact you for further details about scheduling a more in-depth, anonymous interview regarding this topic. (This information will be stored separately from your survey responses and ensure your anonymity after submitting this survey)

29. Contact information: *

____

Thank you for your time!

Unfortunately, this research aims to study adolescents and will only take responses from those under the age of 18. Your time was greatly appreciated. Please email the researcher if you have any additional questions [redacted]
