# Supplementary material for: Social communities in gacha gaming and adolescent problem gambling risk: a mixed methods study
Source: Front Psychiatry. 2026 Oct 2;17:1907023. doi: 10.3389/fpsyt.2026.1907023 (PMC13674343; doi:10.3389/fpsyt.2026.1907023)
Supplement: Supplementary file 2 [file DataSheet2.pdf]

Appendix B

Interview Questions

1. Which gacha game(s) do you play the most? Do you tend to spend money on these games? If so, are they the ones you spend the most on?
2. Are you part of any online or in-game communities related to this gacha game (such as Discord servers, subreddits, group chats, or social media pages)? If so, which ones?
3. How often do you interact with these communities (daily, weekly, occasionally)?
4. Do you feel that being part of these communities influences how you think about spending money in the game? Why or why not?
5. Can you describe a time when something you saw in a community (posts, videos, other players' pulls) made you more interested in spending?
6. How do you feel when you see other players share their gacha pulls or spending experiences?
7. Do community discussions ever make spending feel more normal, expected, competitive, or pressured?
8. Do events like shared hype, banners, or limited-time characters discussed in communities affect your desire to spend?
9. Do you feel that these communities benefit you more positively, negatively, or both when it comes to spending? Explain your perspective.
10. Do you have anything else to share about your experience with gacha social communities and why you choose to spend money on gacha?
