# Supplementary material for: Social communities in gacha gaming and adolescent problem gambling risk: a mixed methods study
Source: Front Psychiatry. 2026 Oct 2;17:1907023. doi: 10.3389/fpsyt.2026.1907023 (PMC13674343; doi:10.3389/fpsyt.2026.1907023)
Supplement: Supplementary file 3 [file DataSheet3.pdf]

Appendix C

Subreddit name	Date posted
r/Genshin_Impact	February 7, 2026
r/ProjectSekai	January 18, 2026
r/WutheringWaves	January 18, 2026
r/MonsterStrike	January 18, 2026
r/battlecats	January 24, 2026
r/BlueArchive	January 24, 2026
r/CookieRunKingdoms	January 18, 2026
r/PuzzleAndDragons	January 18, 2026
r/arknights	January 18, 2026
r/afkarena	February 7, 2026
r/CookierunKingdom	January 18, 2026
r/limbuscompany	January 24, 2026
r/ZZZ_Official	February 7, 2026
r/GenshinImpact	February 7, 2026
